# Mind the (research) gap: a retrospective observational study on the utilization of new medical technologies and related research activities in German hospitals

**DOI:** 10.1186/s12961-025-01342-8

**Published:** 2025-05-30

**Authors:** Tanja Rombey, Helene Eckhardt, Susanne Felgner, Marie Dreger, Alessandro Campione, Hanna Ermann, David Ehlig, Hendrikje Rödiger, Dimitra Panteli, Cornelia Henschke

**Affiliations:** 1 Department of Health Care Management, Technische Universität Berlin, Berlin, Germany; 2 Department of Health Care Management and Berlin Centre for Health Economics Research (BerlinHECOR), Technische Universität Berlin, Berlin, Germany; 3 Chair of Health Economics, Policy and Management, School of Medicine (Med-HSG), Universität St. Gallen, St. Gallen, Switzerland; 4 Institute of General Practice and Interprofessional Care, University Hospital Tübingen, Tübingen, Germany

## Abstract

**Objectives:**

Hospitals play a major role in generating clinical evidence on new medical technologies. Thus far, the extent of German hospitals’ contribution to the evidence base has not been sufficiently investigated. This study aims to: (1) examine the utilization of new medical technologies in German hospitals and its relationship to different hospital characteristics; (2) investigate the participation of German hospitals in research on these technologies and the association between hospital characteristics and research involvement; and (3) investigate the contribution of German hospitals to international research activities, including the levels of evidence of any studies conducted.

**Methods:**

Using a systematically derived sample of 13 new medical technologies and various data sources, we retrospectively analyzed the utilization of and research activities by German hospitals between 2005 and 2017 and explored which hospital characteristics they were associated with. The data were analyzed descriptively and are expressed as bar plots, box plots, quartiles, and crude odds ratios (ORs).

**Results:**

The proportion of German hospitals using new technologies while also being involved in related clinical research was relatively low (ranging from 0.3% to 29.4%, except for transcatheter aortic valve implantation (TAVI), with 60.7%), particularly for prospective studies. Research involvement was positively associated with university hospital status, larger bed capacity, and public ownership. Overall, the research involving German hospitals predominantly consisted of single-arm studies and not randomized controlled trials (RCTs).

**Conclusions:**

Our study identified a gap between hospitals using new medical technologies and their involvement in evidence generation. This imbalance can contribute to uncertainty regarding the actual efficacy, effectiveness and safety of new medical technologies. To ensure evidence-based patient care, it is therefore essential to strengthen the link between research and practice, in both directions. A first step to achieve this could entail restricting the use of new medical technologies to specialized innovation centers (e.g., university hospitals, specialized hospitals) during the initial years of their utilization to ensure an adequate evidence base is generated before widespread implementation.

**Supplementary Information:**

The online version contains supplementary material available at 10.1186/s12961-025-01342-8.

## Introduction

Healthcare systems around the world must balance limited resources with growing health expenditures [1]. Medical technologies, such as pharmaceuticals, medical devices and procedures, are considered among the main drivers of rising healthcare costs [2]. At the same time, they have the potential to increase benefits by improving patient outcomes [3]. However, the rapid pace of innovation, particularly for medical devices, has been accompanied by uncertainties regarding the efficacy, effectiveness and safety of new technologies. While efficacy studies assess whether an intervention, such as a medical technology used in a procedure, achieves its intended effect under ideal, controlled conditions (“Can it work?”), effectiveness studies evaluate whether this effect translates to routine healthcare settings (“Does it work in practice?”). Both also consider safety by assessing the balance between benefits and potential harm in their respective contexts [4, 5].

For a long time, the regulatory requirements for placing medical devices on the market in the European Union (EU) on the basis of a Conformité Européenne (CE) marking were less stringent compared with those in the United States [6]. CE marking for medical devices prioritized market access speed over robust clinical evidence, leading to higher safety risks compared with the U.S. Food and Drug Administration (FDA) approval process [7]. Recalls and withdrawals of certain types of medical devices in Europe [8–12] raised concerns regarding the sufficiency of these requirements and led to a revision in the regulatory framework, resulting in the Medical Device Regulation (MDR, Regulation (EU) 2017/745), which entered into force on 25 May 2017. The MDR was initially set to become fully applicable on 26 May 2021 but was extended by 1 year by the European Commission. This regulation increases requirements for clinical investigation and evaluation, surveillance and transparency [13, 14].

At the same time, even if evidence on the safety and efficacy of medical devices is available at the time of CE marking, real-world utilization of these technologies may show different safety and effectiveness results due to a multitude of factors, such as a broader patient population, longer observation periods or the learning curves of clinicians. For instance, after the market introduction of metal-on-metal hip implants, real-world studies based on registry data reported high rates of implant failure, metal poisoning and revision surgeries [15–17]. For health systems, it is crucial to identify and use only technologies with acceptable effectiveness and safety profiles to optimize both patient care and the allocation of limited resources. However, in many European countries, the inclusion of new technologies into the benefit baskets of statutory health systems and their reimbursement does not necessarily depend on their demonstrated efficacy and/or effectiveness [18]. While countries have been introducing mechanisms to enable evidence-based decisions, such as formal health technology assessments, these are more frequently applied to pharmaceuticals compared with procedures incorporating medical devices and may generally be impeded by the lack of evidence at the point of market entry. To address the latter issue, approaches linking coverage to the generation of post-market evidence, originally implemented as “coverage with evidence development (CED)” by Medicare in the United States, have been adopted in some European countries, including England, France, Germany, the Netherlands, Spain, Sweden and Switzerland [19–21].

German hospitals are legally allowed to use any new medical technology that has obtained a CE marking and has not been explicitly excluded by the Federal Joint Committee (FJC), the highest decision-making body in the German healthcare system [22]. Usually, hospitals in Germany are reimbursed for medical technology as part of diagnosis-related group (DRG) payments, that is, flat rates per case. Problems arise for new high-priced medical technologies, which are not accounted for by existing DRGs. To bridge this gap, hospitals in Germany are allowed to request additional extrabudgetary innovation payments, known as NUB payments, named after the German acronym for New Diagnostic and Treatment Methods (Neue Untersuchungs- und Behandlungsmethoden, Sect. 6(2) KHEntgG, Hospital Payment Act) [23] to cover procedures that incorporate such new technologies. To that end, each hospital must apply annually for innovation payments at the Institute for the Hospital Remuneration System (InEK) before reimbursement prices can be negotiated with health insurance funds; these price negotiations take place during the budget negotiation between each hospital and the statutory health insurance funds [24]. InEK’s approval is valid for 1 year, requiring hospitals to reapply for innovation payments every year if the technology is not yet included in the supplementary payments list or the G-DRG classification. Once this is achieved, there are two types of supplementary payments for medical technologies that cannot appropriately be implemented into the DRG classification: negotiable supplementary payments allow hospitals to negotiate payments with statutory health insurance funds without prior InEK approval, while fixed supplementary payments have a nationally fixed reimbursement rate determined by InEK, allowing for remuneration without prior negotiation. While both types of supplementary payments are part of annual budget negotiations between a hospital and health insurance funds and are therefore subject to negotiated volume thresholds, innovation payments are not part of these annual budget negotiations [25].

For only a few certain procedures involving new high-risk medical devices, hospitals seeking innovation payments are legally required to provide the FJC with available scientific evidence regarding the procedure and the device [26], typically supported by the respective medical device manufacturers. Following a period of 4 weeks that allows other hospitals and manufacturers to submit additional documents, the FJC decides within 3 months whether the benefit of the method is sufficiently proven. If the harmfulness or ineffectiveness of the method can be regarded as sufficiently proven, the FJC decides on the rapid exclusion of the service from inpatient services. If neither the benefit nor the harmfulness or ineffectiveness of the method using the medical device can be considered sufficiently proven, the FJC decides on a trial within 6 months to gather further evidence using a “coverage with evidence development” approach [20]. Apart from this pathway, which only applies to a few procedures on the basis of high-risk devices for which hospitals request innovation payments, there is currently no systematic post-market assessment of the comparative (cost-)effectiveness of new medical technologies to support decisions regarding their inclusion in or removal from the benefit basket of statutory health insurance for inpatient care in Germany. As such, the current regulatory framework does not incentivize German hospitals to generate evidence on many new medical technologies, and the extent to which they contribute to the international evidence base is unclear.

Germany is one of the largest producers of medical devices in Europe, with large investments in research and development. It further represents an important target market for medical devices from elsewhere, with *n* = 1526 regular hospitals in 2022 (excluding hospitals with exclusively psychiatric, psychotherapeutic, neurological and/or geriatric wards and pure day or night clinics) and approximately 16 million inpatient cases in these hospitals [27]. Since many new medical devices are used in the context of inpatient procedures, the willingness and ability of hospitals to serve study sites plays a fundamental role in evidence generation. However, conducting clinical studies requires substantial funding, human resources, equipment, and knowledge. This means that some hospitals might be better placed to participate in research than others, with university hospitals possibly better equipped with research infrastructure and resources compared with other hospitals [25].

Previous studies have explored the association between hospital characteristics and the adoption and utilization of new medical technologies [25, 28]. While evidence regarding the relationship between hospital ownership and the uptake of new technologies is mixed [25, 29, 30], hospital specialization seems to be positively correlated with the utilization of advanced technologies [31]. However, less is known about the link between hospital characteristics and research activity. We hypothesize that larger hospitals, which typically possess more complex resources such as advanced technical equipment and specialized staff, are also more likely to engage in research activities. Similarly, university hospitals are expected to exhibit a greater commitment to clinical research due to their academic mission and focus on innovation.

The overarching aim of our study was to investigate which German hospitals participated in research on the safety, efficacy or effectiveness of new medical technologies and to what extent. Specifically, we aimed to:

1. Identify and characterize German hospitals that used a systematically derived sample of new medical technologies between 2005 and 2017 including variations in using new medical technologies by hospital characteristics;
2. Investigate the participation of German hospitals in research on these technologies and the association between hospital characteristics and research involvement;
3. Examine the contribution of German hospitals to international research activities, with a particular focus on the levels of evidence provided by these research studies.

## Methods

### Study design and setting

**Fig. 1:**
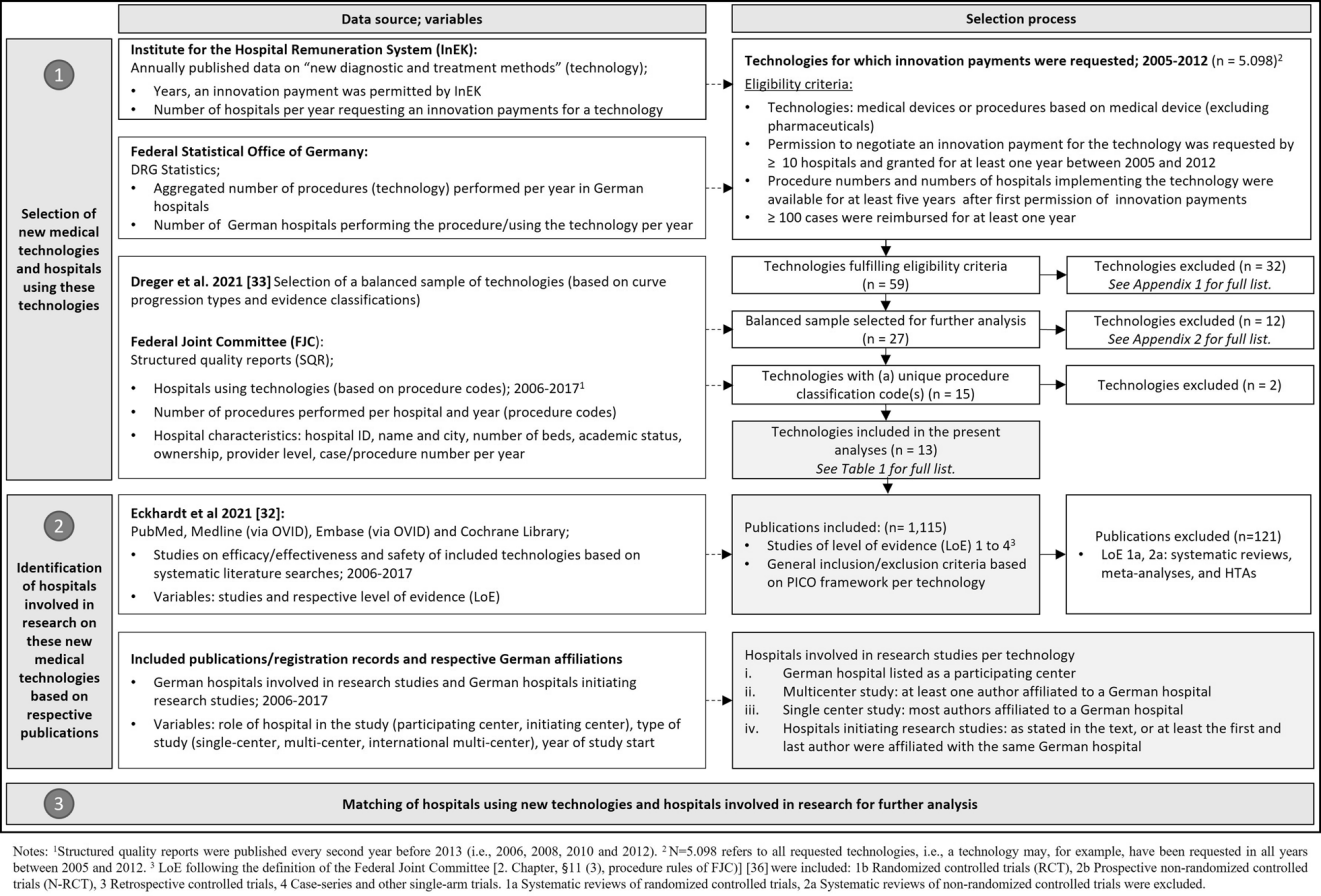
Flow chart of the data sources, variables and selection process

We conducted a retrospective observational study of German hospitals and their utilization of and research on new medical technologies between 2005 and 2017. New medical technologies included diagnostic or treatment procedures relying on a medical device for which permission to negotiate innovation payments was granted at least once before 2017. The data were collected in 2019 and 2020 (Further information can be found here [32]). The processes of technology selection, identification of evidence on efficacy or effectiveness and safety and identification of German hospitals involved in the research (and compared with an international context) are shown in Fig. 1.

### Data selection

#### Selection of new medical technologies and hospitals utilizing these technologies

The present study was part of a project investigating the relationship between published evidence on safety and efficacy or effectiveness and the use of new medical technologies by German hospitals. The selection process for the sample of new medical technologies has been described in detail elsewhere [33] and is summarized in Fig. 1.

In brief, new medical technologies were selected on the basis of the following criteria: first, we identified new technologies (a) for which innovation payments were permitted for at least 1 year between 2005 and 2012 and (b) that were requested by at least 10 hospitals, using a database published annually by the InEK. The time window was chosen to ensure that for all technologies, a sufficient observation period of at least 5 years was available between the first permission of an innovation payment and the latest available data year (2017) at the time of investigation; this leads to variable observation periods across technologies (Table 2). We only included those technologies that had been used in ≥ 100 cases for at least 1 year, identified through DRG statistics, an aggregated dataset of anonymized hospital claims data available from the Research Data Center of the Federal Statistical Office and Statistical Offices of the Federal States upon formal request and respective agreement [34]. For this study, aggregated data on the number of inpatient procedures per technology and year and the number of utilizing hospitals per year were provided by the funder. In total, 59 technologies were grouped empirically on the basis of adoption curve progression. A balanced sample of 27 technologies was selected for further analysis [33] (excluded technologies: Additional file 1, Appendix 1). Of those, only technologies for which a single procedure code (OPS) (as opposed to a combination of codes) is used for claims documentation could be included in the hospital-level analyses (12 excluded technologies: Additional file 1, Appendix 2).

To identify those German hospitals that used the technologies of interest, the structured quality reports (SQRs) of German hospitals were searched for the technologies’ procedure codes. German hospitals are legally required to transparently report performed diagnostic or treatment procedures as well as certain quality data on diagnosis and treatment, on an annual basis, to the FJC, which publishes all SQRs [35]. Until 2012, SQRs were published biennially and have since been published annually. Consequently, we were able to include nine reporting years (2006, 2008, 2010 and 2012–2017). The DRG statistics database that was used for the selection of technologies could not be used for this purpose because it does not facilitate the identification of individual hospitals. However, we compared the annual frequency of procedures and the number of utilizing hospitals for each technology from the DRG statistics and the SQRs for consistency. We excluded technologies for which data on the total number of procedures by all hospitals in the respective observation period deviated by more than 30% as an indicator of limited data quality.

#### Identification of hospitals involved in research on new medical technologies

To identify the hospitals involved in research on the efficacy, effectiveness and safety of the technologies included, we used the results of the evidence mapping performed in a previous stage of our research [32]. For each included technology, the PubMed, Medline (via OVID), Embase (via OVID) and Cochrane Library databases were searched systematically between May and September 2019. In addition, supplementary searches were performed in reference lists of included systematic reviews, in clinical trial registries (clinicaltrials.org, WHO International Clinical Trials Registry Platform (ICTRP)) and in Health Technology Assessment (HTA) databases. The results were screened and categorized using systematic literature review methods; the approach is described in detail elsewhere [32]. For the present analysis, we only included publications of studies with evidence level Ib (randomized controlled trials), IIb (prospective controlled trials), III (retrospective controlled trials) and IV (single-arm trials) as per the definition of the FJC [36]. Systematic reviews, meta-analyses and health technology assessment reports (level Ia or IIa) were excluded but were screened to identify additional individual studies not captured by the systematic search.

For each publication per technology, we assessed which German hospitals had been involved in the study, that is, who had recruited and treated patients with the new technology as part of the study. To that end, we first checked the publications and/or registration records of the studies for information on the study location. If no German hospital was named as the study location, we checked the authors’ affiliations. We assumed that a German hospital was involved in a study when (i) it was listed as a participating center, (ii) it was the affiliation of at least one author for multicenter studies or (iii) it was the affiliation of most authors for single-center studies. When the first and last authors were affiliated with the same German hospital, we assumed that the study was initiated by this hospital if not stated otherwise.

#### Matching utilizing hospitals and hospitals involved in the research

We matched the list of hospitals using a new medical technology under consideration to the hospitals involved in research on this technology by hospital name and city. Furthermore, we grouped publications into studies by trial identifier (i.e. a registration number) or the name of the study to avoid overestimation of hospital research activity by number of publications. If this information was not available in the publications, we compared the publications’ study location, recruitment period and number of included patients to determine whether two or more publications belonged to the same study.

### Data extraction

The data were extracted by one person and verified by a second person in the study group. The following data were extracted from the SQRs for each hospital: hospital ID, hospital name, city, number of beds, university hospital (yes/no), ownership (not-for-profit, private-for-profit, and public), specialty hospital^1^ (yes/no), and number of procedures per year. For data protection reasons, the exact number of procedure codes was not reported in the SQRs if there were fewer than 4 per hospital. In these cases, we imputed a value of 1.

From the study publications with German hospital involvement, we extracted the following information for each hospital and technology: trial identifier or/and name, role of hospital in the study (participating center, initiating center), type of study (single-center, multicenter, international multicenter), year of recruitment start, and study level of evidence (randomized controlled trial, prospective controlled trial, retrospective controlled trial, single-arm trial).

### Outcomes

The primary outcome was the share of hospitals involved in research on a technology out of all hospitals using the respective technology overall and per year. Secondary outcomes included (i) the share of procedures performed in hospitals involved in research and per year, (ii) the strength of the association between hospital characteristics and research involvement and (iii) the share of publications involving German hospitals of all publications identified for the respective technology as part of the systematic literature searches.

### Statistical methods

The data were analyzed descriptively using SPSS Statistics 27 (IBM) and R Studio, version 4.0.2. The categorical data are expressed as absolute numbers and proportions, and the continuous data are expressed as the means and standard deviations (SDs) in box plots. To quantify the associations between hospital characteristics and research involvement, we calculated crude odds ratios (ORs) with 95% confidence intervals (95% CIs). For non-dichotomous characteristics (e.g., ownership), we calculated the odds ratios for hospitals with one expression of this characteristic (e.g., public ownership) versus all other expressions of that variable (e.g., not-for-profit and private-for-profit). The number of hospital beds was dichotomized (< versus ≥ mean number of beds across all hospitals using the technology).

## Results

### Included technologies

A total of 13 technologies (Table 1) were included in the analysis following the selection process (Fig. 1). Technology abbreviations are used throughout the manuscript and further information regarding the technologies can be found in [33].

**Table 1:** Overview of the technologies included (*n* = 13)

Abbreviation	Technology*	Procedure classification codes
ACT	Adjustable continence therapy	5–596.7
BVS	Bioresorbable vascular scaffold in coronary vessels	8-83d.0
DES-LLV	Implantation of a drug-eluting stent in lower leg vessels	8–841.[x]c
DES-ULV	Implantation of a drug-eluting stent in upper leg vessels	8–841.[x]s, 8–841.[x]t; Until 2016: 8–841.[x]b
EABO	Endoaortic balloon occlusion with extracorporeal circulation	8–851.01, 8–851.11, 8–851.31, 8–851.41, 8–851.51
FD-ULV	Flow-diverter (hemodynamically effective implant for endovascular treatment of peripheral aneurysms) in upper leg vessels	8-84b.[x]s, 8-84b.[x]t
LVRC	Lung volume reduction by inserting of coils	5–339.8
MRD	Molecular monitoring of residual tumor burden	1–991
MVAC	Mitral valve annuloplasty with clamp	5-35a.2
PECLA/iLA	Pumpless extracorporal lung assist/interventional lung assist	8–852.2
pVAD	Percutaneous ventricular assist device (microaxial blood pump)	8–839.42, 8–839.43
SE-BMS	Self-expanding bare metal stents in coronary vessels	8-83d.1
TAVI	Transcatheter aortic valve implantation	5-35a.0

^*^Two of these technologies, namely, anticoagulation with citrate during dialysis (OPS 8–854.3, 8–854.5, 8–854.7, 8–855.4, 8–855.6, 8–855.8) and dialysis with high cutoff dialysis membrane (OPS 8–854.8), were excluded due to poor data quality compared with the DRG statistics

### Hospitals’ utilization of new medical technologies and involvement in research

Due to the different dates of first CE-marking of the devices underpinning the included procedures, there was considerable variation in the number of data years in the observation period per technology (Table 2). The number of hospitals using technology ranged from 65 (SE-BMS, 2014–2017) to 468 (DES-ULV, 2008–2017) (Table 2). The total number of procedures performed ranged from 217 (SE-BMS, 2014–2017) to 96,028 (TAVI, 2008–2017), while the mean number of procedures per hospital ranged from 3 ± 6 (FD-ULV, 2012–2017) to 857 ± 814 (TAVI, 2008–2017). Overall, between 0.3% (ACT, 2008–2017) and 60.7% (TAVI, 2008–2017) of the hospitals using a technology were also involved in clinical research on that technology (Table 2). For six technologies (PECLA/iLA, PVAD, DES-LLV, PVAD6, TAVI and DES-ULV), the proportion was considerably higher in the early years of use, while for others, it remained constant over time (Additional file 1, Appendix 3). The overall proportion of procedures that were performed in hospitals involved in clinical research ranged from 0.2% (ACT, 2008–2017) to 87.3% (TAVI, 2008–2017). The trends in procedures performed over time were similar to those of hospital numbers: for six technologies, the proportions were higher in the early years of use (PECLA/iLA, PVAD, DES-LLV, PVAD6, TAVI, and MVAC), while they remained constant over time for the other seven technologies (Additional file 1, Appendix 3a and b).

**Table 2:** Technology utilization and research involvement of hospitals by technology

Technology	Observation period	Utilizing hospital [*N*]	Hospitals involved in research [*N* (%)]	Procedure numbers of utilizing hospitals [*N* (mean ± SD)]	Procedure numbers of research hospitals [*N* (%); mean ± SD]
ACT	2008–2017	385	1 (0.3)	5746 (14.9 ± 33.3)	14 (0.2); 14 ± 0
BVS	2013–2017	357	76 (21.3)	23,259 (65.2 ± 137.3)	14,358 (61.7); 188.9 ± 231.2
DES-LLV	2008–2017	389	9 (2.3)	10,566 (27.2 ± 123.6)	2586 (24.5); 287.3 ± 641.3
DES-ULV	2008–2017	468	10 (2.1)	9327 (19.9 ± 62.3)	1789 (19.2); 178.9 ± 262.7
EABO	2006–2017	91	3 (3.3)	75,733 (832.2 ± 1638.3)	4633 (6.1); 1544.3 ± 1301.6
FD-ULV	2012–2017	143	2 (1.4)	409 (2.9 ± 6.0)	17 (4.2); 17 ± 0*
LVRC	2012–2017	125	8 (6.4)	5233 (41.9 ± 131.3)	1829 (35.0); 228.6 ± 391.4
MRD	2008–2017	194	23 (11.9)	21,859 (112.7 ± 259.8)	9850 (45.1); 428.3 ± 537.8
MVAC	2008–2017	69	4 (5.8)	855 (12.4 ± 19.7)	77 (9.0); 19.3 ± 1.9
PECLA/iLA	2006–2017	255	14 (5.5)	1827 (7.2 ± 14.3)	566 (31.0); 40.4 ± 42.4
pVAD	2006–2017	199	20 (10.1)	6533 (33.2 ± 50.3)	1131 (17.3); 62.8 ± 61.3
SE-BMS	2014–2017	65	15 (23.1)	217** (4.3 ± 16.4)	1 (0.5)*; 1 ± 0**
TAVI	2008–2017	116	68 (58.6)	96,028 (857.4 ± 813.8)	83,862 (87.3); 1310.3 ± 789.0

*ACT* adjustable continence therapy, *BVS* bioresorbable vascular scaffold in coronary vessels, *DES-LLV* implantation of a drug-eluting stent in lower leg vessels, *DES-ULV* implantation of a drug-eluting stent in upper leg vessels, *EABO* endoaortic balloon occlusion with extracorporeal circulation, *FD-ULV* flow-diverter (hemodynamically effective implant for endovascular treatment of peripheral aneurysms) in upper leg vessels, *LVRC* lung volume reduction by insertion of coils, *MRD* molecular monitoring of residual tumor burden, *MVAC* mitral valve annuloplasty with clamp, *PECLA/iLA* pumpless extracorporal lung assist/interventional lung assist, *pVAD* percutaneous ventricular assist device (microaxial blood pump), *SE-BMS* self-expanding bare metal stents in coronary vessels, *TAVI* transcatheter aortic valve implantation

^*^Data refer to only one hospital. One hospital of the total of two hospitals participating in studies could not be identified by searching the structured quality reports

^**^Data refer to only 1 hospital, as 14 of the 15 hospitals involved in studies no longer used the method as of the introduction of OPS in 2014

### Hospital characteristics and respective technology utilization

The characteristics of hospitals using new medical technologies are presented in Table 3. The proportion of public hospitals was highest (42.0% (FD-ULV) to 63.8% (MVAC)), followed by the proportion of not-for-profit hospitals (except for EABO and TAVI). The mean number of beds of hospitals using the technologies ranged from 574 ± 493 (DES-ULV) to 978 ± 577 (MVAC). The proportion of university hospitals among the hospitals using technology ranged from 8.3% (ACT) to 44.9% (MVAC) and the proportion of specialty hospitals from 3.4% (ACT) to 23.1% (TAVI).

**Table 3:** Characteristics of hospitals using the technologies

Technology	Hospitals [*N*]	Ownership [%]			No. of beds [mean ± SD]	University hospital [%]	Specialty hospitals[%]
		Public	Not-for-profit	Private-for-profit			
ACT	385	42.9	42.6	14.5	581 ± 514	8.3	3.4
BVS	357	45.9	35.0	19.0	652 ± 506	11.2	5.9
DES-LLV	389	45.0	34.7	20.3	590 ± 505	9.3	6.7
DES-ULV	468	43.6	37.2	19.2	574 ± 493	9.2	7.7
EABO	91	54.9	15.4	29.7	854 ± 590	38.5	23.1
FD-ULV	143	42.0	37.8	20.3	593 ± 578	12.6	6.3
LVRC	125	44.8	40.0	15.2	746 ± 673	18.4	12.8
MRD	194	53.1	34.5	12.4	809 ± 625	19.6	7.2
MVAC	69	63.8	18.8	17.4	978 ± 577	44.9	15.9
PECLA/iLA	255	54.1	31.4	14.5	747 ± 574	12.9	10.6
pVAD	199	52.3	25.6	22.1	781 ± 602	18.1	9.0
SE-BMS	65	43.1	38.5	18.5	656 ± 527	13.8	6.2
TAVI	116	56.0	18.1	25.9	911 ± 720	33.6	21.6

*ACT* adjustable continence therapy, *BVS* bioresorbable vascular scaffold in coronary vessels, *DES-LLV* implantation of a drug-eluting stent in lower leg vessels, *DES-ULV* implantation of a drug-eluting stent in upper leg vessels, *EABO* endoaortic balloon occlusion with extracorporeal circulation, *FD-ULV* flow-diverter (hemodynamically effective implant for endovascular treatment of peripheral aneurysms) in upper leg vessels, *LVRC* lung volume reduction by insertion of coils, *MRD* molecular monitoring of residual tumor burden, *MVAC* mitral valve annuloplasty with clamp, *PECLA/iLA* pumpless extracorporal lung assist/interventional lung assist, *pVAD* percutaneous ventricular assist device (microaxial blood pump), *SE-BMS* self-expanding bare metal stents in coronary vessels, *TAVI* transcatheter aortic valve implantation

The number of hospitals refers to the time period of the technology considered (Table 2).

### Associations between hospital characteristics and research involvement

**Fig. 2:**
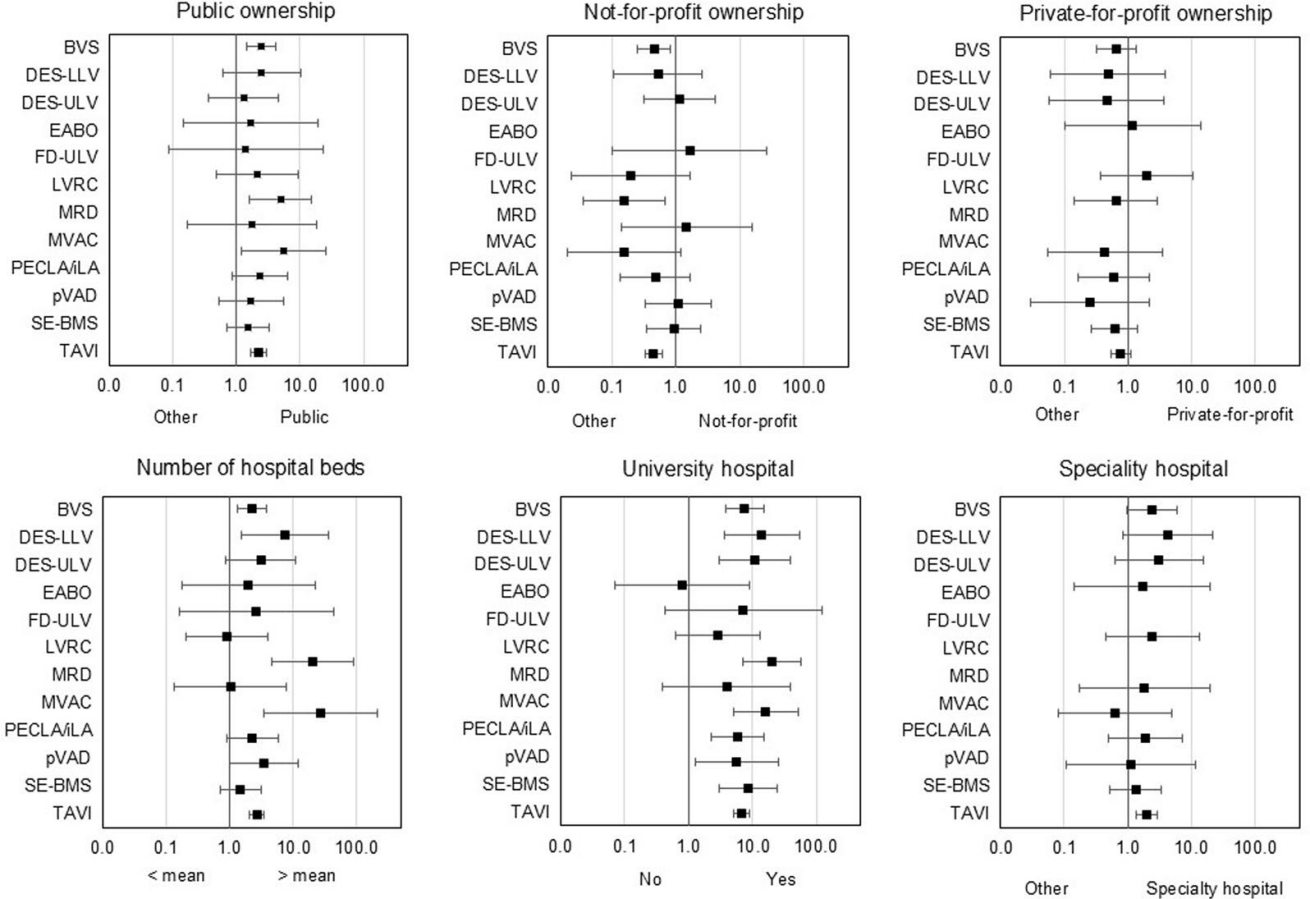
Odds ratios for research involvement by hospital characteristics; 95% confidence intervals

The characteristics of the hospitals stratified by research involvement is shown in Additional file 1, Appendix 4 while in Fig. 2, the odds of being involved in research for hospitals with a certain characteristic are plotted against the odds for hospitals not involved in research but using the technology (numerical values are reported in Additional file 1, Appendix 5).

Public ownership was positively associated with research involvement across all technologies, with significantly higher ORs for three technologies (BVS, MRD and PECLA/iLA). In line with this, not-for-profit and private-for-profit ownership were negatively associated with research involvement for most technologies, with significantly lower ORs for two technologies for not-for-profit hospitals (BVS and MRD). Having more beds than average among all hospitals using the technology was positively associated with research involvement for most technologies, with significantly higher ORs for five technologies (BVS, DES-LLV, MRD, PECLA/iLA, SE-BMS). University hospital status was positively associated with research involvement across all technologies except EABO, with significantly higher ORs for eight technologies (BVS, DES-LLV, DES-ULV, MRD, PECLA/iLA, pVAD, SE-BMS, TAVI). Except for PECLA/iLA, specialty hospitals were also positively associated with research involvement, although the odds were not significantly higher for any technology.

### Share of publications and characteristics of studies involving German hospitals

The number of publications on clinical research per technology involving German hospitals ranged from 1 (ACT) to 144 (TAVI) (Table 4). The number of studies involving German hospitals per technology represented between 3.8% (ACT) and 84.6% (PECLA/iLA) of all study publications on these technologies identified in the systematic literature searches. The number of individual studies with German involvement ranged from 1 (ACT) to 95 (TAVI). Between 25% (MVAC, SE-BMS) and 100% (PECLA/iLA, ACT, MRD, FD-ULV) of all studies with German hospital involvement were also initiated by German hospitals. Between 0% (ACT) and 100% (MRD, DES-ULV, SE-BMS) of the studies involving German hospitals were multicenter studies, of which up to 100% (DES-ULV) were international multicenter studies. The earliest start of a study often occurred well before the observation period (e.g., 1996 for PECLA/iLA).

**Table 4:** Characteristics of studies involving German hospitals

Technology	Observation period	Publication level		Study level				
		Total publications identified (*N*)	Publications involving German hospitals [*N* (%)]	Studies involving German hospitals (*N*)	Studies initiated by German hospitals [*N* (%)]	Multicenter study [*N* (%)]	International multicenter study [*N* (%)]	Start of recruitment [year or span in years]
ACT	2008–2017	26	1 (3.8)	1	1 (100)	0 (0)	0 (0)	2002
BVS	2013–2017	201	37 (18.4)	27	21 (77.8)	18 (66.7)	12 (44.4)	2013–2017
DES-LLV	2008–2017	31	10 (32.3)	7	6 (85.7)	5 (71.4)	3 (42.9)	2005–2011
DES-ULV	2008–2017	41	9 (22.0)	4	2 (50.0)	4 (100)	4 (100)	2001–2013
EABO	2006–2017	16	2 (12.5)	2	1 (50.0)	1 (50.0)	1 (50.0)	1997–1999
FD-ULV	2012–2017	7	3 (42.9)	2	2 (100)	1 (50.0)	0 (0)	2010–2011
LVRC	2012–2017	22	7 (31.8)	6	3 (50.0)	4 (66.7)	4 (66.7)	2008–2012
MRD	2006–2017	68	7 (10.3)	5	5 (100)	5 (100)	1 (20.0)	1999–2004
MVAC	2008–2017	7	4 (57.1)	4	1 (25.0)	3 (75.0)	3 (75.0)	2015
PECLA/iLA	2008–2017	26	22 (84.6)	19	19 (100)	7 (36.8)	2 (10.5)	1996–2011
pVAD	2006–2017	73	12 (16.4)	12	7 (58.3)	8 (66.7)	6 (50.0)	2000–2015
SE-BMS	2014–2017	15	9 (60.0)	4	1 (25.0)	4 (100)	3 (75.0)	2009–2011
TAVI	2008–2017	461	144 (31.2)	95	79 (83.2)	38 (40.0)	28 (29.5)	2005–2016

*ACT* adjustable continence therapy, *BVS* bioresorbable vascular scaffold in coronary vessels, *DES-LLV* implantation of a drug-eluting stent in lower leg vessels, *DES-ULV* implantation of a drug-eluting stent in upper leg vessels, *EABO* endoaortic balloon occlusion with extracorporeal circulation, *FD-ULV* flow-diverter (hemodynamically effective implant for endovascular treatment of peripheral aneurysms) in upper leg vessels, *LVRC* lung volume reduction by insertion of coils, *MRD* molecular monitoring of residual tumor burden, *MVAC* mitral valve annuloplasty with clamp, *PECLA/iLA* pumpless extracorporal lung assist/interventional lung assist, *pVAD* percutaneous ventricular assist device (microaxial blood pump), *SE-BMS* self-expanding bare metal stents in coronary vessels, *TAVI* transcatheter aortic valve implantation

The level of evidence of studies involving German hospitals is presented in Table 5. For most technologies, the studies consisted of single-arm trials (level IV), ranging from 49.5% (TAVI) to 100% (ACT, EABO, FD-ULV) of all studies. The proportion of randomized controlled trials (level Ib) ranged from 0% (ACT, EABO, FD-ULV, MVAC) to 49.5% (DES-ULV), while the highest number of RCTs (*n* = 4) was found for TAVI.

**Table 5:** Studies with German participation according to the level of evidence per technology

Technology	Level of evidence				Total [*N*]
	Ib [*N* (%)]	IIb [*N* (%)]	III [*N* (%)]	IV [*N* (%)]	
ACT	0 (0%)	0 (0%)	0 (0%)	1 (100%)	1
BVS	1 (3.7%)	0 (0%)	6 (22.2%)	20 (74.1%)	27
DES-LLV	3 (42.9%)	0 (0%)	0 (0%)	4 (57.1%)	7
DES-ULV	2 (50%)	0 (0%)	0 (0%)	2 (50%)	4
EABO	0 (0%)	0 (0%)	0 (0%)	2 (100%)	2
FD-ULV	0 (0%)	0 (0%)	0 (0%)	2 (100%)	2
LVRC	1 (16.7%)	0 (0%)	0 (0%)	5 (83.3%)	6
MRD	1 (20%)	1 (20%)	0 (0%)	3 (60%)	5
MVAC	0 (0%)	1 (25%)	0 (0%)	3 (75%)	4
PECLA/iLA	1 (5.3%)	0 (0%)	6 (31.6%)	12 (63.2%)	19
pVAD	2 (16.7%)	0 (0%)	2 (16.7%)	8 (66.7%)	12
SE_BMS	1 (25%)	1 (25%)	0 (0%)	2 (50%)	4
TAVI	4 (4.2%)	7 (7.4%)	37 (38.9%)	47 (49.5%)	95
Total	16	10	51	111	188

*ACT* adjustable continence therapy, *BVS* bioresorbable vascular scaffold in coronary vessels, *DES-LLV* implantation of a drug-eluting stent in lower leg vessels, *DES-ULV* implantation of a drug-eluting stent in upper leg vessels, *EABO* endoaortic balloon occlusion with extracorporeal circulation, *FD-ULV* flow-diverter (hemodynamically effective implant for endovascular treatment of peripheral aneurysms) in upper leg vessels, *LVRC* lung volume reduction by insertion of coils, *MRD* molecular monitoring of residual tumor burden, *MVAC* mitral valve annuloplasty with clamp, *PECLA/iLA* pumpless extracorporal lung assist/interventional lung assist, *pVAD* percutaneous ventricular assist device (microaxial blood pump), *SE-BMS* self-expanding bare metal stents in coronary vessels, *TAVI* transcatheter aortic valve implantation

LoE following the definition of the FJC 2. Chapter, §11 (3), procedure rules of FJC (24) were included: Ib randomized controlled trials (RCT), IIb prospective non-randomized controlled trials (N-RCT), III retrospective controlled trials, IV case-series and other single-arm trials. Ia systematic reviews of randomized controlled trials and IIa systematic reviews of non-randomized controlled trials were not included.

## Discussion

To our knowledge, this is the first study to systematically investigate the research activities on new medical technologies in German hospitals. For 13 medical technologies, the number of hospitals engaging in research activities on clinical evidence generation and the utilization of new medical technologies were examined. Particular attention was given to variations regarding hospital characteristics and the relationship between hospital characteristics and research participation. Furthermore, the contribution of German hospitals to international clinical research efforts was investigated with an emphasis on the evidence level of studies involving German hospital participation.

During the observation period and for different technologies, the number of hospitals using a new medical technology ranged between 65 and 468, representing 4.1–29.4% of all regular hospitals in Germany (*n* = 1592) [37]. Among hospitals using a specific technology, participation in related clinical research varied widely, from 0.3% for ACT (2008–2017) to 60.7% for TAVI (2008–2017). However, for most technologies included in this analysis, less than 12% of the hospitals using them were also involved in research, with higher rates of research involvement for only three technologies, all from the field of cardiovascular surgery (21.3% for BVS, 29.4% for SE-BMS, and 60.7% for TAVI).

The share of hospitals using new technology that also participated in clinical trials on this technology tended to decrease over time. Overall, the early years of utilization showed higher research participation for six technologies, with declining research engagement over time potentially reflecting consolidated evidence, as observed with TAVI [32]. This decline can also be partly explained by the diffusion of new technologies to other hospitals. However, when evidence of effectiveness is insufficient, the number of non-research hospitals using new technology should not increase. Furthermore, it is important to critically address the potential risks associated with the widespread adoption of new medical technologies, particularly in hospitals with low procedure volumes. Such diffusion can dilute expertise and compromise the quality of care, especially when the supporting evidence for these technologies is limited. Centralization of services could enhance outcomes by concentrating experience and resources, a consideration that is particularly important for technologies based on novel procedures, by enabling the early identification and mitigation of potential risks before broader implementation. The initial use of new medical technologies in specialized (innovation) centers not only enhances the safety and efficacy of procedures, but coupled with the appropriate mechanisms for evidence generation, can also lay the groundwork for evidence-based expansion to other hospitals [38].

Our results also show that hospitals involved in research usually treated more patients with these technologies than other hospitals, particularly in the first year(s) of use when uncertainty regarding safety, efficacy and effectiveness is generally higher [39, 40]. For most of the new technologies studied (such as pVAD, DES-LLV and DES-ULV), the number of procedures performed increased over time, potentially implying that these technologies replaced others. However, we also found that the absolute number of procedures for some new technologies (e.g., PECLA/iLA) decreased, and hospitals involved in clinical trials “moved away” from technology use faster than non-researching hospitals. Taking into account that research participation was measured at the point of publication, this finding could be limited due to publication times. However, it supports the hypothesis that researching hospitals might be more likely to abandon technologies with unconvincing evidence profiles, ultimately providing better care.

Regarding the analysis of hospital characteristics, the largest proportion of hospitals using new medical technologies were public hospitals, although public hospitals accounted for only 28.8% of general hospitals in Germany in 2017 [37]. University hospitals, which are predominantly public hospitals, accounted for 2% of all general hospitals in 2017 [37]. Nevertheless, the reasons for varying levels of utilization may differ depending on specific technology. For instance, the lower engagement of for-profit hospitals in performing TAVI may seem surprising at first glance, as TAVI could potentially serve as a marketing tool to attract additional patients. From a system perspective, a consensus paper by the German Cardiac Society (DGK) published in 2014 outlined that hospitals should perform at least 50 TAVI implantations annually [41] to offer the procedure reliably. In 2015, the FJC issued a guideline that defines structural and personnel requirements as well as measures to ensure process quality [42]. These requirements may not have been easy to meet for all private hospitals. What is more, for for-profit hospitals, efficiency considerations play an important role. Implementing TAVI requires investment in equipment and training, which may increase the costs and thus the potential revenues, while reimbursement for this complex procedure is moderate [43]. For-profit hospitals, however, focus on procedures with high profit margins.

Characteristics positively associated with research involvement included public ownership, above-average number of beds and university hospital status. These factors are often interconnected, as university hospitals are typically publicly owned and have higher bed capacities. Legally mandated to engage in teaching and research, university hospitals generally possess superior research infrastructure and resources compared with other hospitals [25], such as clinical trial centers staffed with study nurses, statisticians, administrative personnel and dedicated ethics committees. Additionally, physicians in university hospitals may receive protected research time through clinician–scientist programs, facilitating tasks such as grant writing and investigator-initiated trials [44, 45]. Similarly, specialized hospitals play a crucial role in conducting clinical trials, primarily due to their advanced resources and expertise. Nevertheless, internationally coordinated teams that pool their resources and knowledge to collaborate on clinical research is a sine qua non in research on efficacy and effectiveness of new medical technologies. Here, the European Clinical Research Infrastructure Network (ECRIN) serves as the primary point of contact [46].

Concerning the contribution of German hospitals to international research activities, study involvement by number of publications varied widely across the included technologies, ranging from 3.8% (ACT) to 84.6% (PECLA/iLA), with the latter being a technology developed in Germany. Furthermore, the identified research activity in German hospitals mainly comprised studies of evidence levels III (27%) and IV (59%), which is consistent with previous findings that many studies on new technologies are based on uncontrolled study designs [32]. The German Federal Science Council has already denounced that the potential of clinical trials is not being fully exploited in German hospitals, despite the wide recognition of their importance [47]. Our results confirm this issue.

Overall, our study identified a dearth of high-quality evidence for several technologies and underlines the need for conducting high-quality studies to enable evidence-based decision-making for patients, physicians, hospitals, insurers and policymakers. Most studies were uncontrolled and therefore not suitable for (comparative) effectiveness assessment. Research on medical technologies is challenging in terms of study designs and statistical methods. This is attributable to device-specific characteristics (e.g., device–operator interaction), problems regarding the blinding process, possible organizational and economic implications for providers or the problem of distinguishing the extent to which observed benefits are attributable to the diagnostic or treatment component of certain patient pathways [48]. Healthcare systems should provide advice for manufacturers within setting up studies regarding comparators, study designs, etc., or at least provide guidelines. At the EU level, the new Health Technology Assessment Regulation foresees joint scientific consultations for technology developers [49], and the EMA has been piloting a program specifically for high-risk devices [50].

If RCTs are not feasible, real-world data to evaluate comparative effectiveness may be used but require ensuring adequate data quality and avoiding common sources of bias [51, 52]. Depending on the stage in the life cycle of medical technologies, guidance on appropriate methodologies, such as those recommended by the IDEAL (Idea, Development, Exploration, Assessment and Long-term Study) framework [52], may help to set up studies for medical technologies during each stage in the life cycle of medical technology. Particularly regarding the long-term evaluation of the potential benefits and risks of medical technologies, studies based on real-world data (administrative data, registry data and other observational data) are receiving increasing attention [53]. Notably, the focus should not be on the sheer number of studies but rather on their quality. A high volume of publications does not necessarily correlate with robust evidence on technology’s benefits.

The lack of mandatory evidence-based assessments prior to reimbursement can expose patients to unnecessary risks, including potential harm or lack of benefit, while also contributing to inefficiencies in the healthcare system [32]. Countries such as France and the United Kingdom have established such approaches to assess comparative effectiveness of new technologies, including medical devices, prior to reimbursement [54, 55]. Given the time lag from study initiation to publication – often three, four, or more years – approaches such as coverage with evidence development offer a pragmatic solution for evaluating technologies prior to widespread adoption without delaying patient access. This approach can also allow for de-implementation when negative results emerge. Although Germany introduced the potential for a CED evaluation process in 2016 (§137 h SGB V) for certain new diagnostic and treatment methods on the basis of a high-risk medical device in inpatient care, there are only limited results so far. Only a few CED studies have been initiated or are in the process of being set up [56]. Nevertheless, in 2019 with the amendments to Sect. 137e of the German Social Code, Book V (SGB V), the legislator has been stipulating that the FJC will fully finance the costs for the scientific monitoring and evaluation of trial projects itself, a shift from previous regulations where companies had to contribute to overhead costs to an appropriate extent. Despite these changes, completing studies on time remains challenging due to factors such as patient recruitment. Further efforts are necessary to foster (international) multicenter studies and clinical research infrastructures [57, 58] and to strengthen knowledge gained from real-world data [51].

Clinical trials, especially RCTs, require a high level of personnel and financial resources, limiting their execution to large (university) hospitals. Sufficient yet not overly complex bureaucratic structures are required that will enable appropriate hospitals to participate in evidence generation [33]. The introduction of the Medical Research Act in Germany in October 2024 marks an important step toward accelerating clinical trials while maintaining high patient safety standards [59]. Sustained political commitment to a comprehensive health research strategy, including proactive management and system coordination, are key facilitators [60]. Such a strategy should also ensure that robust new knowledge is made available to hospitals in a timely and systematized manner [33]. This is important to address, as previous research has shown that in the current system, the quality of scientific evidence does not always influence decisions on the adoption of innovations in healthcare [61]. Managers even tend to prioritize implementation and cost evidence from their own or other hospital organizations over scientific evidence [62].

Beyond the process of adoption, “real-world” evidence should be generated through medical device registries, which could further enhance the evaluation of effectiveness and risks under routine conditions during the diffusion process, including evidence on mid- and long-term outcomes [33]. And finally, the value of technology must be rewarded accordingly. Therefore, it is essential to link the identified benefits transparently to the reimbursement and to reward innovation, but also to de-implement medical technologies from the market if necessary. Although first concepts of CED have emerged, there is still no systematic approach linking benefits with reimbursement for medical technologies in inpatient care in Germany.

### Limitations

Although we used a systematically derived sample of new technologies [33] and our analyses were based on comprehensive data sources, there are certain limitations that should be considered. First, the sample of technologies might not be representative of all new technologies, as we had to exclude half of the technologies from the larger sample. Second, procedure volumes for the analyzed technologies were extracted from hospital documentation on the basis of the respective procedure codes. Therefore, the data quality depended on the coding practices of the individual hospitals. Additionally, for data protection reasons, exact values are not reported in the SQRs if there are fewer than four procedures per hospital. As we imputed a value of 1 procedure in those situations, the total procedure numbers were likely slightly underestimated.

The rapid review methodology used to identify available evidence per technology also has some limitations [32]. Since publications in languages other than English or German were excluded, the share of publications with German study involvement is most likely an overestimation. Furthermore, unpublished studies were not considered, potentially distorting the proportion of hospitals participating in research, as there is evidence that many studies remain unpublished years after study completion [63]. However, only published studies can contribute to the evidence base, and researchers are required by the Declaration of Helsinki to make the results of their research on human subjects publicly available [64].

## Conclusions

This retrospective observational study highlights the discrepancy between the rapid utilization of new medical technologies in German hospitals and their participation in generating the scientific evidence supporting these technologies. It provides an analysis of the clinical research engagement of German hospitals for 13 new medical technologies and its contribution to the overall clinical evidence base. Although many hospitals use new technologies, their involvement in accompanying clinical studies, especially in the early phases of use, lags behind both in terms of volume and level of evidence. This imbalance can lead to uncertainties regarding the actual efficacy, effectiveness and safety of new medical technologies. To ensure evidence-based patient care, it is therefore essential to strengthen the link between research and practice, in both directions. A first step to achieve this could entail restricting the use of new medical technologies to specialized innovation centers (e.g., university hospitals, specialized hospitals) during the initial years of their utilization to ensure an adequate evidence base is generated before widespread implementation. Policymakers should therefore create an environment that supports systematic evidence generation and that can inform evidence-based decisions.

## Supplementary Information

Additional file 1.

## Data Availability

The data gathered and analyzed during the study can be made available upon reasonable request. In addition, we used structured quality reports (SQRs) of German hospitals between 2005 and 2017 to identify those German hospitals that used the technologies of interest by searching for technologies’ procedure codes. Structured quality reports reporting the number of procedures of technologies of interest are available here: https://www.g-ba.de/themen/qualitaetssicherung/datenerhebung-zur-qualitaetssicherung/datenerhebung-qualitaetsbericht/.

## Abbreviations

ACT: Adjustable continence therapy
BVS: Bioresorbable vascular scaffold in coronary vessels
CE: Conformité Européenne
CIs: Confidence intervals
e.g.: For example
DES-LLV: Implantation of a drug-eluting stent in lower leg vessels
DES-ULV: Implantation of a drug-eluting stent in upper leg vessels
EABO: Endoaortic balloon occlusion with extracorporeal circulation
EU: European Union
FD-ULV: Flow-diverter (hemodynamically effective implant for endovascular treatment of peripheral aneurysms) in upper leg vessel
FJC: Federal Joint Committee (German: Gemeinsamer Bundesausschuss, G-BA)
DRG: Diagnosis-related group
HTA: Health Technology Assessment
InEK: Institute for the Hospital Remuneration System (German: Institut für das Entgeltsystem im Krankenhaus)
LVRC: Lung volume reduction by insertion of coils
MRD: Molecular monitoring of residual tumor burden
MVAC: Mitral valve annuloplasty with clamp
OPS: Procedure codes (German: Operationen- und Prozedurenschlüssel)
ORs: Odds ratios
PECLA/iLA: Pumpless extracorporal lung assist/interventional lung assist
pVAD: Percutaneous ventricular assist device (microaxial blood pump)
SD: Standard deviation
SE-BMS: Self-expanding bare metal stents in coronary vessels
SQRs: Structured quality reports
TAVI: Transcatheter aortic valve implantation
USA: United States of America
vs: Versus

## Footnotes

1 Specialty hospitals include hospitals focusing on the treatment of specific diseases, groups of diseases or even a particular organ. Unlike general hospitals, specialized hospitals do not provide comprehensive care across a wide range of medical fields but instead concentrate on one or a few specific areas of specialization. For the structured quality reports until 2015, there was a variable that defined whether the hospital is of type specialiy hospital. We extrapolated the values for this variable until 2017.
