# Supplementary material for: Mind the (research) gap: a retrospective observational study on the utilization of new medical technologies and related research activities in German hospitals
Source: Health Res Policy Syst. 2025 May 30;23:72. doi: 10.1186/s12961-025-01342-8 (PMC12124022; doi:10.1186/s12961-025-01342-8)
Supplement: Supplementary file 1 — Additional file 1. [file 12961_2025_1342_MOESM1_ESM.docx]

# **SUPPLEMENTARY MATERIAL**

# **Appendix 1: List of excluded technologies (n = 32)**

| **Technology** |
| --- |
| Antibody coated coronary stent |
| Baroreceptor activation |
| Bioactive extra-long coils for intracranial aneurysm therapy |
| Coronary bifurcation stents |
| Coronary stent, self-expanding (at least two stents, drug-eluting) |
| Defibrillator with subcutaneously implantable electrode |
| Double J metal stent for urinary diversion in ureteral strictures |
| Drug-coated balloon catheter in artificial vessels |
| Drug-coated balloon catheter in lower arm vessels |
| Drug-coated balloon catheter in other vessels |
| Drug-coated balloon catheter in shoulder and upper arm vessels |
| Drug-coated balloon catheter in thoracic vessels |
| Drug-coated balloon catheter in visceral vessels |
| Drug-eluting stents for the treatment of lesions of the supraaortic arteries |
| Endobronchial valve |
| Endovascular implantation/repair of a stent prosthesis using an endostapler |
| Esophageal sphincter implant, magnetic |
| Ex vivo chemosensitivity testing |
| Extra-long coils (3D) for intracranial aneurysm therapy |
| Fenestrated endoprostheses for abdominal aortic aneurysms |
| Flow-diverter (Hemodynamically effective implant for endovascular treatment) in intracranial vessels |
| Hybrid cochlear implant |
| Insertion of coated (covered) stents with bioactive surface for peripheral vessels |
| Insertion of coated (covered) stents with bioactive surface for visceral and supraaortic vessels |
| Intraaneurysmal hemodynamically effective implant for endovascular treatment of intracranial aneurysms. |
| Minimally invasive heart valve surgery (endovascular implantation of pulmonary valve replacement) |
| Minimally invasive operations on heart valves (implantation of a mitral valve replacement) |
| Neurostimulator for stimulation of the spinal cord or peripheral nervous system, rechargeable |
| Percutaneous transluminal clipping for mitral valve regurgitation |
| Therapy of scoliosis by means of magnetic-controlled rods |
| Vertical Expandable Prosthetic Titanium Rib |
| Volume coils for intracranial aneurysm therapy |

**Appendix 2: Further excluded technologies due to combination of procedure codes (n = 12)**

| **Technology** | **Procedure classification codes** |
| --- | --- |
| Drug-coated balloon catheter in abdominal vessels | 8-836.09 (since 2017: 8-836.0q or 8-836.0r) and 8-83b.b |
| Drug-coated balloon catheter in intracranial vessels | 8-836.00 and 8-83b.b |
| Drug-coated balloon catheter in lower leg vessels | 8-836.0c and 8-83b.b |
| Drug-coated balloon catheter in upper leg vessels | 8-836.0b (since 2017 8-836.0s or 2017 8-836.0t) and 8-83b.b |
| Drug-coated balloon catheter in coronary vessels | 8-837.0 and 8-83b.b0 [since 2011: 8-83b.b*(2-5); since 2013: 8-83b.b*(6-9)] |
| Drug-eluting beads for transarterial chemoembolization | 8-836.k and 8-83b.10 |
| Excimer laser extraction of pacemakers and defibrillator electrodes | 5-378.a0 and 5- 378.1, 5- 378.2, 5- 378.6, or 5-378.7 |
| Cardiac event recorder after ablative measures for atrial fibrillation/ atrial tachycardia | 5-377.8 and 8-835 |
| Fluorescence-assisted transurethral resection | 5-573.41 or 1-661 and 5-989 |
| Intracranial endovascular thrombectomy (microwire retriever) | 8-836.80 and 8- 83b.80, 8- 83b.82, or 8-83b.83 |
| Bioactive coils for intracranial aneurysm therapy | 8-836.m0 and 8- 83b.31 |
| Fetoscopic drainage therapy | 5-754.1 (OPS not unique to this technology only) |

## **Appendix 3a: Development of hospital numbers and case volume over time**

**Appendix 3b: Development of hospital numbers and case volume over time**

|  | **2006** | **2008** | **2010** | **2012** | **2013** | **2014** | **2015** | **2016** | **2017** | **Time trend (%)** |
| --- | --- | --- | --- | --- | --- | --- | --- | --- | --- | --- |
| **Technology** | **Number of hospitals involved in clinical trials/number of all utilizing hospitals (%)** | | | | | | | | | |
| ACT | - | 1/121 (1%) | 0/179 (0%) | 1/162 (1%) | 0/161 (0%) | 0/153 (0%) | 1/138 (1%) | 1/134 (1%) | 1/139 (1%) | → |
| BVS | - | - | - | - | 52/167 (31%) | 74/231 (32%) | 70/248 (28%) | 68/233 (29%) | 54/175 (31%) | → |
| DES-LLV | - | 4/33 (12%) | 7/70 (10%) | 8/150 (5%) | 5/161 (3%) | 7/175 (4%) | 5/204 (2%) | 4/198 (2%) | 6/228 (3%) | ↓ |
| DES-ULV | - | 6/40 (15%) | 7/105 (7%) | 8/190 (4%) | 7/201 (3%) | 9/216 (4%) | 7/227 (3%) | 10/243 (4%) | 10/264 (4%) | ↓ |
| EABO | 3/60 (5%) | 3/66 (5%) | 3/68 (4%) | 3/76 (4%) | 3/75 (4%) | 2/73 (3%) | 3/76 (4%) | 3/72 (4%) | 3/72 (4%) | → |
| FD-ULV | - | - | - | 0/47 (0%) | 1/38 (3%) | 1/43 (2%) | 0/31 (0%) | 1/25 (4%) | 1/38 (3%) | → |
| LVRC | - | - | - | 6/34 (18%) | 7/59 (12%) | 7/77 (9%) | 8/79 (10%) | 8/77 (10%) | 6/61 (10%) | ↓ |
| MRD | - | 17/68 (25%) | 20/94 (21%) | 23/112 (21%) | 23/117 (20%) | 23/113 (20%) | 22/116 (19%) | 23/116 (20%) | 23/122 (19%) | → |
| MVAC | - | 2/11 (18%) | 1/17 (6%) | 1/12 (8%) | 0/11 (0%) | 3/17 (18%) | 2/24 (8%) | 4/38 (11%) | 3/42 (7%) | → |
| PECLA/iLA | 5/21 (24%) | 12/67 (18%) | 11/120 (9%) | 11/116 (9%) | 11/109 (10%) | 10/91 (11%) | 11/96 (11%) | 7/71 (10%) | 6/54 (11%) | ↓ |
| PVAD | 7/12 (58%) | 10/29 (34%) | 11/38 (29%) | 6/27 (22%) | 7/42 (17%) | 9/63 (14%) | 12/80 (15%) | 12/115 (10%) | 14/179 (8%) | ↓ |
| SE-BMS | - | - | - | - | - | 0/44 (0%) | 0/37 (0%) | 1/34 (3%) | 0/19 (0%) | → |
| TAVI | - | 25/44 (57%) | 30/86 (35%) | 31/92 (34%) | 31/93 (33%) | 32/99 (32%) | 32/92 (35%) | 32/88 (36%) | 33/85 (39%) | ↓ |
| **Number of procedures performed in hospitals involved in clinical trials/ number of procedures in all utilizing hospitals (%)** | | | | | | | | | | |
| ACT | - | 2/609 (0%) | 0/1247 (0%) | 4/793 (1%) | 0/730 (0%) | 0/710 (0%) | 2/649 (0%) | 3/515 (1%) | 3/493 (1%) | → |
| BVS | - | - | - | - | 3367/5,112 (66%) | 4731/7,395 (64%) | 3724/6,270 (59%) | 1997/3,527 (57%) | 539/955 (56%) | → |
| DES-LLV | - | 210/323 (65%) | 172/386 (45%) | 337/1,315 (26%) | 296/1,166 (25%) | 308/1,318 (23%) | 311/1,742 (18%) | 443/2,094 (21%) | 509/2,222 (23%) | ↓ |
| DES-ULV | - | 56/118 (47%) | 27/219 (12%) | 244/1,692 (14%) | 174/913 (19%) | 147/1,082 (14%) | 298/1,310 (23%) | 397/1,854 (21%) | 446/2,139 (21%) | → |
| EABO | 296/6,036 (5%) | 418/7,074 (6%) | 358/8,113 (4%) | 438/9,184 (5%) | 520/9,059 (6%) | 606/8,580 (7%) | 642/9,023 (7%) | 729/9,080 (8%) | 626/9,584 (7%) | → |
| FD-ULV | - | - | - | 0/95 (0%) | 8/71 (11%) | 6/64 (9%) | 0/62 (0%) | 1/53 (2%) | 2/64 (3%) | → |
| LVRC | - | - | - | 280/404 (69%) | 437/865 (51%) | 409/1,190 (34%) | 265/1,374 (19%) | 310/916 (34%) | 128/484 (26%) | ↓ |
| MRD | - | 582/1,422 (41%) | 894/2,015 (44%) | 1,019/2,505 (41%) | 1,338/2,867 (47%) | 1,477/3,086 (48%) | 1,477/3,383 (44%) | 1,591/3,363 (47%) | 1,472/3,218 (46%) | → |
| MVAC | - | 15/33 (45%) | 8/219 (4%) | 4/64 (6%) | 0/38 (0%) | 9/63 (14%) | 6/123 (5%) | 20/147 (14%) | 15/168 (9%) | ↓ |
| PECLA/iLA | 46/74 (62%) | 123/237 (52%) | 56/291 (19%) | 115/324 (35%) | 66/256 (26%) | 48/200 (24%) | 64/218 (29%) | 41/149 (28%) | 7/78 (9%) | ↓ |
| PVAD | 50/55 (91%) | 41/94 (44%) | 62/112 (55%) | 72/190 (38%) | 72/361 (20%) | 55/605 (9%) | 111/965 (12%) | 270/1,613 (17%) | 398/2,538 (16%) | ↓ |
| SE-BMS | - | - | - | - | - | 0/146 (0%) | 0/37 (0%) | 1/25 (4%) | 0/9 (0%) | → |
| TAVI | - | 1,080/1,385 (78%) | 3,333/5,886 (57%) | 5,472/9,964 (55%) | 5,736/10,876 (53%) | 7,181/13,539 (53%) | 8,780/15,904 (55%) | 9,850/17,897 (55%) | 10,968/20,577 (53%) | ↓ |
| →, constant; ↓, falling | | | | | | | | | | |

**Appendix 4: Characteristics of the hospitals using technologies stratified by research involvement**

| **Technology**  (non-research,  research hospital) | **Hospitals [N]** | **Ownership** | | | **Relative no. of beds  > mean** | **University hospital**  **[%]** | **Specialty**  **hospital [%]** |
| --- | --- | --- | --- | --- | --- | --- | --- |
|  |  | **Public [%]** | **Not-for-profit [%]** | **Private-for-profit [%]** |  |  |  |
| **ACT** | 385 | 42.9% | 42.6% | 14.5% | 34.5% | 8.3% | 3.4% |
| non-research | 384 | 42.7% | 42.7% | 14.6% | 34.4% | 8.1% | 3.4% |
| research | 1 | 100.0% | 0.0% | 0.0% | 100.0% | 100.0% | 0.0% |
| **BVS** | 357 | 45.9% | 35.0% | 19.0% | 35.0% | 11.2% | 5.9% |
| non-research | 281 | 41.3% | 38.4% | 20.3% | 31.0% | 5.7% | 4.6% |
| research | 76 | 63.2% | 22.4% | 14.5% | 50.0% | 31.6% | 10.5% |
| **DES-LLV** | 389 | 45.0% | 34.7% | 20.3% | 32.9% | 9.3% | 6.7% |
| non-research | 380 | 44.5% | 35.0% | 20.5% | 31.8% | 8.2% | 6.3% |
| research | 9 | 66.7% | 22.2% | 11.1% | 77.8% | 55.6% | 22.2% |
| **DES-ULV** | 468 | 43.6% | 37.2% | 19.2% | 33.1% | 9.2% | 7.7% |
| non-research | 458 | 43.4% | 37.1% | 19.4% | 32.5% | 8.3% | 7.4% |
| research | 10 | 50.0% | 40.0% | 10.0% | 60.0% | 50.0% | 20.0% |
| **EABO** | 91 | 54.9% | 15.4% | 29.7% | 50.5% | 38.5% | 23.1% |
| non-research | 88 | 54.5% | 15.9% | 29.5% | 50.0% | 38.6% | 22.7% |
| research | 3 | 66.7% | 0.0% | 33.3% | 66.7% | 33.3% | 33.3% |
| **FD-ULV** | 143 | 42.0% | 37.8% | 20.3% | 28.0% | 12.6% | 6.3% |
| non-research | 141 | 41.8% | 37.6% | 20.6% | 27.7% | 12.1% | 6.4% |
| research | 2 | 50.0% | 50.0% | 0.0% | 50.0% | 50.0% | 0.0% |
| **LVRC** | 125 | 44.8% | 40.0% | 15.2% | 40.0% | 18.4% | 12.8% |
| non-research | 117 | 43.6% | 41.9% | 14.5% | 40.2% | 17.1% | 12.0% |
| research | 8 | 62.5% | 12.5% | 25.0% | 37.5% | 37.5% | 25.0% |
| **MRD** | 194 | 53.1% | 34.5% | 12.4% | 40.7% | 19.6% | 7.2% |
| non-research | 171 | 49.1% | 38.0% | 12.9% | 33.9% | 12.3% | 8.2% |
| research | 23 | 82.6% | 8.7% | 8.7% | 91.3% | 73.9% | 0.0% |
| **MVAC** | 69 | 63.8% | 18.8% | 17.4% | 49.3% | 44.9% | 15.9% |
| non-research | 65 | 63.1% | 18.5% | 18.5% | 49.2% | 43.1% | 15.4% |
| research | 4 | 75.0% | 25.0% | 0.0% | 50.0% | 75.0% | 25.0% |
| **PECLA/iLA** | 255 | 54.1% | 31.4% | 14.5% | 36.1% | 12.9% | 10.6% |
| non-research | 241 | 52.3% | 32.8% | 14.9% | 32.8% | 10.0% | 10.8% |
| research | 14 | 85.7% | 7.1% | 7.1% | 92.9% | 64.3% | 7.1% |
| **pVAD** | 199 | 52.3% | 25.6% | 22.1% | 36.7% | 18.1% | 9.0% |
| non-research | 179 | 50.3% | 26.8% | 22.9% | 34.6% | 14.5% | 8.4% |
| research | 20 | 70.0% | 15.0% | 15.0% | 55.0% | 50.0% | 15.0% |
| **SE-BMS** | 65 | 43.1% | 38.5% | 18.5% | 26.2% | 13.8% | 6.2% |
| non-research | 50 | 40.0% | 38.0% | 22.0% | 20.0% | 8.0% | 6.0% |
| research | 15 | 53.3% | 40.0% | 6.7% | 46.7% | 33.3% | 6.7% |
| **TAVI** | 116 | 56.0% | 18.1% | 25.9% | 47.4% | 33.6% | 21.6% |
| non-research | 48 | 50.0% | 18.8% | 31.3% | 41.7% | 10.4% | 18.8% |
| research | 68 | 60.3% | 17.6% | 22.1% | 51.5% | 50.0% | 23.5% |

**Appendix 5: Odds ratios and 95% confidence intervals for being involved in research of hospitals with a certain characteristic have been plotted against the odds of hospitals without that characteristic**

|  | **Relative no. of beds ≥ mean** | **Private-for-profit ownership** | **Public ownership** | **Non-profit ownership** | **Specialty hospital** | **University hospital** |
| --- | --- | --- | --- | --- | --- | --- |
| BVS | 2.23 (1.331 to 3.735) | 0.665 (0.33 to 1.342) | 2.438 (1.445 to 4.114) | 0.462 (0.256 to 0.833) | 2.425 (0.966 to 6.086) | 7.644 (3.8 to 15.377) |
| DES-LLV | 7.492 (1.534 to 36.599) | 0.484 (0.06 to 3.927) | 2.497 (0.615 to 10.132) | 0.531 (0.109 to 2.59) | 4.238 (0.835 to 21.521) | 14.073 (3.593 to 55.112) |
| DES-ULV | 3.111 (0.865 to 11.19) | 0.461 (0.058 to 3.683) | 1.302 (0.372 to 4.558) | 1.129 (0.314 to 4.059) | 3.118 (0.637 to 15.263) | 11.053 (3.063 to 39.883) |
| EABO | 2 (0.175 to 22.867) | 1.192 (0.104 to 13.731) | 1.667 (0.146 to 19.062) | n.c. | 1.7 (0.146 to 19.733) | 0.794 (0.069 to 9.097) |
| FD-ULV | 2.615 (0.16 to 42.849) | n.c. | 1.39 (0.085 to 22.672) | 1.66 (0.102 to 27.105) | n.c. | 7.294 (0.436 to 122.101) |
| LVRC | 0.894 (0.204 to 3.919) | 1.961 (0.365 to 10.53) | 2.157 (0.492 to 9.449) | 0.198 (0.024 to 1.664) | 2.452 (0.45 to 13.356) | 2.91 (0.643 to 13.174) |
| MRD | 20.457 (4.636 to 90.276) | 0.645 (0.141 to 2.943) | 4.92 (1.607 to 15.065) | 0.155 (0.035 to 0.684) | n.c. | 20.238 (7.177 to 57.069) |
| MVAC | 1.031 (0.137 to 7.769) | n.c. | 1.756 (0.173 to 17.844) | 1.472 (0.141 to 15.411) | 1.833 (0.173 to 19.443) | 3.964 (0.391 to 40.171) |
| PECLA/iLA | 26.658 (3.426 to 207.422) | 0.438 (0.056 to 3.453) | 5.476 (1.2 to 24.992) | 0.158 (0.02 to 1.227) | 0.636 (0.08 to 5.062) | 16.275 (5.043 to 52.527) |
| pVAD | 2.306 (0.907 to 5.864) | 0.594 (0.166 to 2.128) | 2.307 (0.849 to 6.274) | 0.482 (0.135 to 1.717) | 1.929 (0.507 to 7.342) | 5.885 (2.23 to 15.525) |
| SE-BMS | 3.5 (1.025 to 11.956) | 0.253 (0.03 to 2.144) | 1.714 (0.537 to 5.477) | 1.088 (0.334 to 3.541) | 1.119 (0.108 to 11.623) | 5.75 (1.306 to 25.31) |
| TAVI | 1.485 (0.705 to 3.129) | 0.623 (0.27 to 1.438) | 1.519 (0.72 to 3.201) | 0.929 (0.357 to 2.416) | 1.333 (0.533 to 3.333) | 8.6 (3.037 to 24.355) |
| Total | 2.715 (2.088 to 3.531) | 0.764 (0.534 to 1.094) | 2.223 (1.697 to 2.912) | 0.448 (0.326 to 0.617) | 2.014 (1.382 to 2.936) | 6.839 (5.194 to 9.004) |
